# Long‐term exercise in mice has sex‐dependent benefits on body composition and metabolism during aging

**DOI:** 10.14814/phy2.13011

**Published:** 2016-11-14

**Authors:** Rachel C. McMullan, Scott A. Kelly, Kunjie Hua, Brian K. Buckley, James E. Faber, Fernando Pardo‐Manuel de Villena, Daniel Pomp

**Affiliations:** ^1^ Department of Genetics University of North Carolina at Chapel Hill Chapel Hill North Carolina; ^2^ Department of Zoology Ohio Wesleyan University Delaware Ohio; ^3^ Department of Cell Biology and Physiology University of North Carolina at Chapel Hill Chapel Hill North Carolina; ^4^ Lineberger Comprehensive Cancer Center University of North Carolina at Chapel Hill Chapel Hill North Carolina

**Keywords:** C57BL/6J, exercise training, mouse, physical activity, physiological response, voluntary running wheels

## Abstract

Aging is associated with declining exercise and unhealthy changes in body composition. Exercise ameliorates certain adverse age‐related physiological changes and protects against many chronic diseases. Despite these benefits, willingness to exercise and physiological responses to exercise vary widely, and long‐term exercise and its benefits are difficult and costly to measure in humans. Furthermore, physiological effects of aging in humans are confounded with changes in lifestyle and environment. We used C57BL/6J mice to examine long‐term patterns of exercise during aging and its physiological effects in a well‐controlled environment. One‐year‐old male (*n* = 30) and female (*n* = 30) mice were divided into equal size cohorts and aged for an additional year. One cohort was given access to voluntary running wheels while another was denied exercise other than home cage movement. Body mass, composition, and metabolic traits were measured before, throughout, and after 1 year of treatment. Long‐term exercise significantly prevented gains in body mass and body fat, while preventing loss of lean mass. We observed sex‐dependent differences in body mass and composition trajectories during aging. Wheel running (distance, speed, duration) was greater in females than males and declined with age. We conclude that long‐term exercise may serve as a preventive measure against age‐related weight gain and body composition changes, and that mouse inbred strains can be used to characterize effects of long‐term exercise and factors (e.g. sex, age) modulating these effects. These findings will facilitate studies on relationships between exercise and health in aging populations, including genetic predisposition and genotype‐by‐environment interactions.

## Introduction

It has been estimated that Americans older than 65 years‐age will double in number from 40 million in 2010 to 81 million by 2040 (Akerman et al. 2014). During the aging process, individuals generally experience a gain in body fat, redistribution of body fat, increase in intramuscular fat, and a decrease in lean mass and bone density (Zamboni et al. 2005; St‐Onge and Gallagher 2010; Kelly et al. 2011). These concomitant changes in body composition and body mass are thought to be due to alterations in resting metabolic rate (St‐Onge and Gallagher 2010). It is common for energy expenditure to decline due to slower metabolism, decreased lean mass, and less physical activity (Herring et al. 2014). Further, the loss of lean mass during aging may cause dysregulation of energy expenditure via decreased basal metabolic rate (BMR) (Roberts and Rosenberg 2006; Williams and Wood 2006), reduced physical fitness, and lower quality of life (Milanovic et al. 2013). Accompanying alterations in body composition and metabolism, physical activity levels decline with aging in both rodents (Ingram 2000; Turner et al. 2005; Vaanholt et al. 2008) and humans (Bouchard 1990; Caspersen and Merritt 1995) in part due to the changes in skeletal muscle structure and function (Akerman et al. 2014). Meanwhile, chronic health issues, such as obesity and other metabolic and cardiac conditions, increase with age in humans (Pearson et al. 2012).

It is well established that regular aerobic exercise results in beneficial health outcomes including among others, prevention or delay of diseases such as heart disease, stroke, certain forms of cancer, and dementia. In addition, physical activity is effective in weight management, regulation of obesity risk (Booth et al. 2012; Kelly and Pomp 2013; Blundell et al. 2015), and mitigation of physiological changes (e.g. muscle loss) that contribute to decline in exercise capacity with aging (Jacobs et al. 1985; Akerman et al. 2014; Place et al. 2015). Even though much literature suggests that regular exercise in elderly individuals mitigates age‐related decline in health, three‐fourths of older adults do not meet the recommended levels of exercise (Nied and Franklin 2002). Despite the benefits of exercise some individuals do not exercise or fail to experience positive response to exercise treatments (Kelly et al. 2011; Booth et al. 2012; Kelly and Pomp 2013; Blundell et al. 2015; Gordon et al. 2016). Thus, developing a better understanding of the physiological effects of exercise during aging is vital for application to the growing aged human population (Akerman et al. 2014).

Exercise traits (e.g. amount, intensity) vary depending on sex in both humans and rodents. In humans, physical activity traits are greater in males compared to females, whereas, females run further and at greater speeds than males in multiple mouse strains (Lightfoot et al. 2004; Lightfoot 2008). Sex differences exist in body weight, composition, and energy metabolism in both humans and rodents. In both humans and rodents, females tend to maintain white adipose tissue and resist loss of these energy stores, compared to males. Human females have greater adipose mass, greater brown adipose mass, store adipose in different regions, have lower lean mass and greater circulating free fatty acids (FFA) than males. Even with these sex differences, females maintain glucose homeostasis in part because estrogen (E2) concentrations are tightly regulated between puberty and menopause (Mauvais‐Jarvis 2015). In both sexes, E2 regulates adipose development and deposition, body composition, energy balance, mitochondrial function, fatty acid transport and oxidation, and glucose metabolism. Postmenopausal women display a loss of E2, which leads to alterations in metabolism, body weight gain, and increased visceral fat in both humans and rodents (Zarins et al. 2009; Kim 2014; MacDonald et al. 2015; Palmer and Clegg 2015).

Habitual low‐intensity exercise (over 100 days) results in cardiorespiratory and metabolic adaptations, specifically the loss of fat mass and maintenance of fat‐free mass (Bouchard 1990). However, there is a relative lack of knowledge regarding the effects of long‐term exercise (exercise > 3 months) or the effects of exercise initiated during midlife. Animal models provide a time‐efficient alternative to longitudinal human studies, which are difficult and expensive to conduct. However, most rodent studies have examined the impact of habitual exercise for short periods [6 to ~60 days (Takeshita et al. 2012)] in young mice (typically 60 days old). Previous studies on long‐term exercise in mice demonstrated positive outcomes on median lifespan, maintenance of motor coordination, and altered expression of genes involved in heart and immune function (Bronikowski et al. 2003; Turner et al. 2005; Garcia‐Valles et al. 2013). However, there is a gap in knowledge regarding long‐term exercise patterns during aging, the metabolic response to long‐term exercise, and the effect of sex on these traits in a controlled environment.

In this report, we examined long‐term voluntary exercise patterns in an aging mouse population of C57BL/6J mice. The present results represent data collected within a larger experiment aiming to understand the impact of exercise, beginning at mid‐life and extending through the aging process, in protection against disease. The C57BL/6J strain was selected because it is the most widely used in biomedical research, is the mouse reference genome (Didion and de Villena 2013), and is commercially available at mid‐age. The median lifespan for C57BL/6J males is 901 days (30 months) and 866 days (28.8 months) for females (Yuan et al. 2009). We examined physiological response to voluntary exercise in both sexes of C57BL/6J mice starting at ~1 year‐age until ~2 years‐age, which is equivalent to ~40–70 years in humans (Flurkey et al. 2007). We demonstrate that long‐term exercise even with decreasing physical workload provides beneficial outcomes on body weight and composition during aging, and that it does so in a sex‐dependent manner. In addition, our findings on metabolic response to physical activity during aging may aid establishing guidelines for exercise in the aging human population.

## Methods

### Animals

Thirty male and 30 female C57BL/6J mice were purchased from the National Institute of Aging at 1 year of age and after arrival at University of North Carolina‐Chapel Hill were allowed to acclimate to the vivarium for 1 week. Mice were then assessed by Echo MRI for body weight and composition (EchoMRI‐100, Echo Medical Systems, Houston, TX) and placed into individual indirect calorimetry cages (Phenomaster/Labmaster, TSE SYSTEMS, Chesterfield, MO) for 48 h with O_2_ consumption and CO_2_ production, energy expenditure and food and water consumption measured for 48 h. Respiratory exchange rate (RER, VCO_2_/VO_2_) was also calculated.

Following initial body composition and metabolic measures, animals in the experimental cohort were individually housed with access to running wheels (Lafayette Instruments, Lafayette, IN; model 80850L), while animals in the control cohort were single‐housed with access to ordinary lab‐animal enrichment (mouse huts) but no access to running wheels.

Voluntary running was recorded at 1‐min intervals for 23–24 h a day for 12 months. The following daily exercise parameters were obtained: distance (total revolutions), time spent running (cumulative 1‐min intervals in which at least 1 revolution was recorded), average speed (total revolutions/time spent running), and maximum speed (highest number of revolutions in any 1‐min interval within a 24‐h period) (Kelly et al. 2012).

Body weight and composition were measured by MRI every 60 days; these measurements were conducted for all mice on the same day, except as noted below. Animals in both groups were removed from their respective cages for 48 h of indirect calorimetry assessment after 6 months and again after 1 year. At both time points, and again due to limitations in the number of indirect calorimetry cages, MRI measurement of body weight and body composition (obtained just prior to placement in the indirect calorimeters) for all 60 mice required 26 days; the initial 30 mice measured were from the experimental cohort, while the final 30 mice measured were from the control group. Throughout the paper, we refer to the age of the mice as either ~1, ~1.1, ~1.4, ~1.5, ~1.6, ~1.8 or ~2 years representing an approximate age of 372, 423, 520, 549, 606, 669, or 731 days. The approximate age at each phenotype time point was calculated as the mean age in days from the actual age in days of each individual mouse at the time of data collection.

Four mice in the experimental group died during the first 3 months of the experiment. Three of these mice died prior to the measures at ~1.1 years (~423 days) of age and the fourth prior to the measures at ~1.4 years (~520 days) of age. These mice were included in statistical analyses of all measures prior to their death. Upon death, each of these four mice was replaced with an individual previously placed into the control group. Upon replacement and granting of wheel access these mice were placed in the experimental group for all subsequent statistical analyses. This was done to maintain sample size in the experimental cohort, and had no impact on results relative to removal of these mice from the analyses (data not shown). All procedures were approved by and conducted in accordance with the guidelines set forth by the Institutional Animal Care and Use Committee at the University of North Carolina at Chapel Hill.

### Statistical analysis

Estimated marginal means and standard errors of body mass (g), percent body fat and percent lean mass were calculated at the beginning of the experiment at a mean age of ~1 year, and every other month over the next year. Percent body fat (and lean) was calculated as (fat mass/body mass) × 100. Percent change in variables [(pre–post/pre) × 100] was calculated relative to the prior measurement. Estimated marginal means and standard errors of RER were calculated at a mean age of ~1, ~1.5, and ~2 years of age. Measures represent diurnal (lights on) means on day‐2 of a 2‐day trial. Home cage activity was monitored for two consecutive days and mean activity levels were analyzed. General Linear Models (GLM) [Univariate GLM analysis of variance (ANOVA) (SPSS, Chicago, IL)] were utilized to examine the effects of sex (fixed effect), wheel access (fixed effect), and the sex‐by‐wheel access interaction on all phenotypic measurements. Statistical significance was defined as *P *<* *0.05, and all *P*‐values presented are two‐tailed. GLM for data collected from TSE equipment also included the following covariates in the analysis: Activity, mean of the diurnal home cage activity on day‐2 of the 2‐day trial; Batch, reflects group that individuals were tested in within a given time point; VO_2_ (mL/kg/h), VCO_2_ (mL/kg/h), and RER values represent the diurnal (lights on) means on day‐2 of the 2‐day trial. Additionally, for a subset of the phenotypes, repeated measures ANOVAs [GLM (SPSS, Chicago, IL)] were utilized to investigate the effects of age across all groups (sex and experimental vs. control).

Estimated marginal means and standard errors were calculated for the physical activity traits (mean revolutions per day, mean time spent running per day, mean running speed per day). Measures were represented as means across 57 weeks. Comparisons between sexes were analyzed, using GLM (SPSS, Chicago, IL). Wheel freeness was included in models as a covariate. Wheel freeness was calculated as the number of wheel revolutions following acceleration to a given velocity. Pearson partial correlations (*r*; controlling for sex) were calculated for revolutions/day (distance), 1‐min intervals/day (time, cumulative 1‐min intervals in which at least one revolution was recorded), and average running speed (total revolutions/time spent running) and metabolic traits at ~1, ~1.5, and ~2 years. Pearson partial correlations (*r*; controlling for sex and wheel access) were calculated for physical activity traits and body composition traits at ~1, ~1.1, ~1.4, ~1.5, ~1.6, ~1.8, ~2 years. Pearson partial correlations (*r*; controlling for sex and wheel access) were calculated for body composition and metabolic traits at ~1, ~1.5, ~2 years. Degrees of freedom ranged from 20 to 54.

## Results

For simplicity, we first summarize data for metabolic changes across aging in control mice (no wheel access) to establish “baseline” phenotypes, and then describe experimental data across time for mice provided wheel access in the following sections: (1) patterns of exercise, (2) impact of exercise on metabolic changes, and (3) exercise‐by‐sex interactions on metabolic changes.

### Age and sex contribute to metabolic changes in control mice

**Figure 1:**
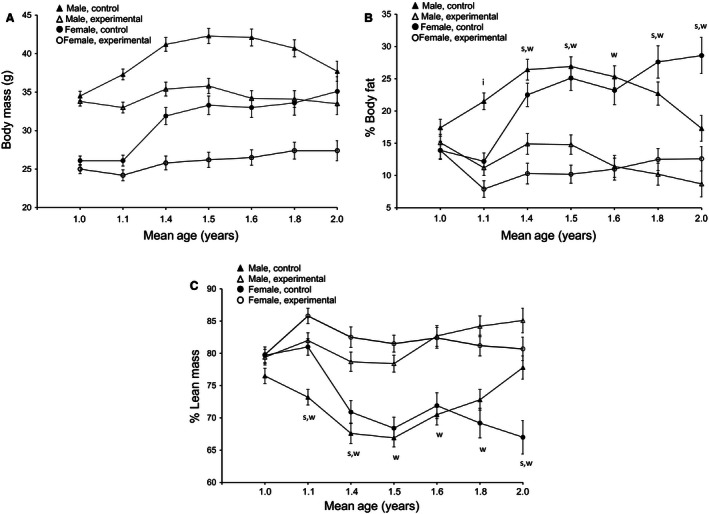
Estimated marginal means and standard errors of (A) body mass (g), (B) percent body fat, and (C) percent lean mass beginning at approximately one year of age and extending over the course of the following year. Wheel access (experimental) or no wheel access (control) was granted after the measurement at ~1 year of age. (A) At all time points, General Linear Models (GLM) revealed that males weighed significantly more than females (*P* < 0.05) and, with the exception of age ~1 year (immediately prior to wheel access), wheel access significantly reduced mass (*P* < 0.05). No significant sex‐by‐wheel access interactions were detected. However, at ~1.1 years of age, following the first 51 days of wheel access, the sex‐by‐wheel access interaction (*F*_1, 52_ = 3.354; *P* = 0.073) suggested that wheel access reduced body mass to a greater extent among male mice. For panels (B) and (C), at a given mean age, an “i” indicates a significant (*P* < 0.05) interaction, a “s” indicates a significant effect of sex, and a “w” indicates a significant effect of wheel access on percent body fat.

The body mass of control C57BL6/J mice increased during aging from ~1 to ~2 years, and body mass changes occurred in a sex‐dependent manner (Fig. 1A). Over the entire period studied, males weighed significantly (*P* < 0.001) more than females (Table 1, Table S1, Fig. 1A). Additionally, there were significant sex effects on body mass changes between measurement time points except from ~1.5 to ~1.8 years (*P* < 0.05, Fig. 1A, Table 1). In control male mice, we observed an initial increase in body mass (~1 to ~1.4 years), stable body mass levels in the midsection of our study (~1.4 to ~1.6 years) and a decline in body mass at older ages (~1.6 to ~2 years). In contrast, in control female mice, body mass remained stable early (~1 to ~1.1 years) and then increased for the remainder of the study (~1.1 to ~2 years) (Fig. 1A, Table S1).

**Table 1:** Effects of sex (male vs. female) and exercise (wheel vs. no wheel) on body composition. Measures were taken beginning at approximately one year of age and extended over the course of the following year

Trait	*N*	Sex	Wheel access	Interaction
~Year 1				
Body mass (g)	59	*F*_1, 55_ = 199.689 ***P *** **< 0.001**	*F*_1, 55_ = 2.127 *P *= 0.150	*F*_1, 55_ = 0.094 *P *= 0.760
% Fat	59	*F*_1, 55_ = 3.092 *P *= 0.084	*F*_1, 55_ = 0.767 *P *= 0.385	*F*_1, 55_ = 0.749 *P* = 0.390
% Lean	59	*F*_1, 55_ = 2.174 *P* = 0.146	*F*_1, 55_ = 1.400 *P* = 0.242	*F*_1, 55_ = 1.349 *P* = 0.250
~Year 1.1				
Body mass (g)	56	*F*_1, 52_ = 213.576 ***P*** ** < 0.001**	*F*_1, 52 _= 20.102 ***P*** ** < 0.001**	*F*_1, 52_ = 2.911 *P* = 0.094
% Fat	56	*F*_1, 52_ = 25.098 ***P*** ** < 0.001**	*F*_1, 52_ = 33.781 ***P*** ** < 0.001**	*F*_1, 52_ = 5.575 ***P*** ** = 0.022**
% Lean	56	*F*_1, 52_ = 22.356 ***P*** ** < 0.001**	*F*_1, 52_ = 30.530 ***P*** ** < 0.001**	*F*_1, 52_ = 2.750 *P* = 0.103
% Change in Mass	55	*F*_1, 51_ = 7.896 ***P*** ** = 0.007**	*F*_1, 51_ = 25.754 ***P*** ** < 0.001**	*F*_1, 51_ = 1.769 *P* = 0.189
% Change in % Fat	55	*F*_1, 51_ = 10.332 ***P*** ** = 0.002**	*F*_1, 51_ = 32.134 ***P*** ** < 0.001**	*F*_1, 51_ = 1.313 *P* = 0.257
% Change in % Lean	55	*F*_1, 51_ = 18.918 ***P*** ** < 0.001**	*F*_1, 51_ = 24.674 ***P*** ** < 0.001**	*F*_1, 51_ = 1.840 *P* = 0.181
~Year 1.4				
Body mass (g)	54	*F*_1, 50_ ^=^ 97.594 ***P*** ** < 0.001**	*F*_1, 50_ = 38.151 ***P*** ** < 0.001**	*F*_1, 50_ = 0.040 *P* = 0.842
% Fat	54	*F*_1, 50 _= 6.494 ***P*** ** = 0.014**	*F*_1, 50_ = 51.438 ***P*** ** < 0.001**	*F*_1, 50_ = 0.053 *P* = 0.818
% Lean	54	*F*_1, 50 _= 4.942 ***P*** ** = 0.031**	*F*_1, 50_ = 50.711 ***P*** ** < 0.001**	*F*_1, 50_ = 0.042 *P* = 0.839
% Change in Mass	53	*F*_1, 49_ = 7.414 ***P*** ** = 0.009**	*F*_1, 49_ = 37.680 ***P*** ** < 0.001**	*F*_1, 49_ = 16.525 ***P*** ** < 0.001**
% Change in % Fat	53	*F*_1, 49_ = 4.128 ***P*** ** = 0.048**	*F*_1, 49_ = 2.212 *P* = 0.143	*F*_1, 49_ = 8.603 ***P*** ** = 0.005**
% Change in % Lean	53	*F*_1, 49_ = 5.763 ***P*** ** = 0.020**	*F*_1, 49_ = 40.322 ***P*** ** < 0.001**	*F*_1, 49_ = 0.410 *P* = 0.525
~Year 1.5				
Body mass (g)	53	*F*_1, 49_ = 80.383 ***P*** ** < 0.001**	*F*_1, 49_ = 42.233 ***P*** ** < 0.001**	*F*_1, 49_ = 0.073 *P* = 0.789
% Fat	53	*F*_1, 49_ = 4.164 ***P*** ** = 0.047**	*F*_1, 49_ = 74.067 ***P*** ** < 0.001**	*F*_1, 49_ = 0.771 *P* = 0.384
% Lean	53	*F*_1, 49_ = 2.503 *P* = 0.120	*F*_1, 49_ = 71.116 ***P*** ** < 0.001**	*F*_1, 49_ = 0.313 *P* = 0.578
% Change in Mass	52	*F*_1, 48_ = 0.226 *P* = 0.637	*F*_1, 48_ = 2.763 *P* = 0.103	*F*_1, 48_ = 0.449 *P* = 0.506
% Change in % Fat	52	*F*_1, 48_ = 0.135 *P* = 0.715	*F*_1, 48_ = 0.060 *P* = 0.807	*F*_1, 48_ = 0.178 *P* = 0.675
% Change in % Lean	52	*F*_1, 48_ = 2.975 *P* = 0.091	*F*_1, 48_ = 60.021 ***P*** ** < 0.001**	*F*_1, 48_ = 0.258 *P* = 0.614
~Year 1.6				
Body mass (g)	53	*F*_*1*, 49_ = 55.311 ***P*** ** < 0.001**	*F*_*1*, 49_ = 40.734 ***P*** ** < 0.001**	*F*_1, 49_ = 0.417 *P* = 0.521
% Fat	53	*F*_1, 49_ = 0.502 *P* = 0.482	*F*_1, 49_ = 51.737 ***P*** ** < 0.001**	F_1, 49_ = 0.199 *P* = 0.657
% Lean	53	*F*_1, 49_ = 0.091 *P* = 0.764	*F*_1, 49_ = 44.899 ***P*** ** < 0.001**	*F*_1, 49_ = 0.277 *P* = 0.601
% Change in mass	53	*F*_1, 49_ = 6.902 ***P*** ** = 0.011**	*F*_1, 49_ = 0.539 *P* = 0.466	*F*_1, 49_ = 6.539 ***P*** ** = 0.014**
% Change in % Fat	53	*F*_1, 49_ = 8.162 ***P*** ** = 0.006**	*F*_1, 49_ = 0.383 *P* = 0.539	*F*_1, 49_ = 11.721 ***P*** ** = 0.001**
% Change in % Lean	53	*F*_1, 49_ = 0.696 *P* = 0.408	*F*_1, 49_ = 45.924 ***P*** ** < 0.001**	*F*_1, 49_ = 0.023 *P* = 0.880
~Year 1.8				
Body mass (g)	51	*F*_1, 47_ = 31.317 ***P*** ** < 0.001**	*F*_1, 47_ = 27.705 ***P*** ** < 0.001**	*F*_1, 47_ = 0.029 *P* = 0.866
% Fat	51	*F*_1, 47_ = 3.458 *P* = 0.069	*F*_1, 47_ = 51.524 ***P*** ** < 0.001**	*F*_1, 47_ = 0.442 *P* = 0.509
% Lean	51	*F*_1, 47_ = 3.438 *P* = 0.070	*F*_1, 47_ = 43.578 ***P*** ** < 0.001**	*F*_1, 47_ = 0.026 *P* = 0.873
% Change in Mass	51	*F*_1, 47_ = 2.240 *P* = 0.141	*F*_1, 47_ = 7.599 ***P*** ** = 0.008**	*F*_1, 47_ = 0.704 *P* = 0.406
% Change in % Fat	51	*F*_1, 47_ = 13.176 ***P*** ** = 0.001**	*F*_1, 47_ = 1.476 *P* = 0.231	*F*_1, 47_ = 1.226 *P* = 0.274
% Change in % Lean	51	*F*_1, 47_ = 1.306 *P* = 0.259	*F*_1, 47_ = 53.151 ***P*** ** < 0.001**	*F*_1, 47_ = 0.162 *P* = 0.689
~Year 2				
Body mass (g)	49	*F*_1, 45_ = 8.647 ***P*** ** = 0.005**	*F*_1, 45_ = 16.183 ***P*** ** < 0.001**	*F*_1, 45_ = 1.515 *P* = 0.225
% Fat	49	*F*_1, 45_ = 11.882 ***P*** ** = 0.001**	*F*_1, 45_ = 31.040 ***P*** ** < 0.001**	*F*_1, 45_ = 2.794 *P* = 0.225
% Lean	49	*F*_1, 45_ = 13.721 ***P*** ** = 0.001**	*F*_1, 45_ = 25.857 ***P*** ** < 0.001**	*F*_1, 45_ = 2.409 *P* = 0.128
% Change in Mass	49	*F*_1, 45_ = 18.171 ***P*** ** < 0.001**	*F*_1, 45_ = 0.512 *P* = 0.478	*F*_1, 45_ = 12.510 ***P*** ** = 0.001**
% Change in % Fat	49	*F*_1, 45_ = 17.647 ***P*** ** < 0.001**	*F*_1, 45_ = 0.225 *P* = 0.638	*F*_1, 45_ = 0.216 *P* = 0.644
% Change in % Lean	49	*F*_1, 45_ = 10.069 ***P*** ** = 0.003**	*F*_1, 45_ = 35.749 ***P*** ** < 0.001**	*F*_1, 45_ = 3.765 *P* = 0.059

Statistical significance was judged at *P *<* *0.05 (in bold), and all *P*‐values presented are two‐tailed.

Body composition also changed during aging in control mice (Fig. 1B, C). One‐year‐old females had lower body fat and higher lean mass than males, but the situation reversed itself as the mice aged. Males had greater percent body fat and lower percent lean mass than females until ~1.6 years of age when females had greater percent body fat and lower percent lean mass than males. Overall the pattern of increase in body fat in both sexes closely followed the changes in body mass (compare Fig. 1A and B). In females increases occurred over the length of the study, while in males there was initial increase in body fat, followed by a period of stabilization and a final decrease (Fig. 1B). The patterns for lean mass are similar but inverted (Fig. 1A, C). Significant (*P* < 0.05) sex effects on body fat (Table 1, Fig. 1B), lean mass (Table 1, Fig. 1C) and changes in percent fat and lean mass between consecutive measurements were observed for most time points (Table 1). The changes in both percent body fat and percent lean mass during aging in control mice occurred in a sex‐dependent manner (Fig. 1B, C, Table S1).

**Figure 2:**
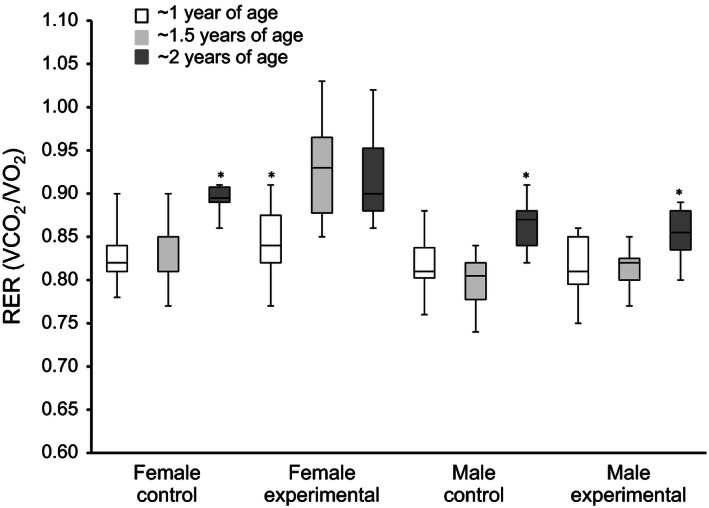
Respiratory exchange ratio during aging and across experimental groups (sex; treatment). Repeated measure analysis of variance (ANOVAs) [GLM (SPSS, Chicago, IL)] revealed a significant effect of age across all groups (sex and experimental vs. control) (*P* < 0.05). Pairwise comparisons indicated that RER at ~ 2 years was significantly higher compared to ~1 year (*P* < 0.001) or ~1.5 years (*P* < 0.001). Additionally, within each treatment group a similar trend was observed ‐ asterisks represent results for within group pairwise comparisons.

Indirect calorimetry was performed three times on all mice: at the beginning of the experiment (~1 year) at ~1.5 years and at ~2 years of age (Table 2, Table S2). Over the period of study, RER levels ranged from 0.78 to 0.88 in control mice (Fig. 2, Table S2). In control males, VO_2_, VCO_2_, and RER levels were lower at ~1 year compared to ~1.5 years and greater at ~1.5 years compared to ~2 years (Table S2, Fig. 2). Female control mice had lower VO_2_, VCO_2_ and RER levels at ~1 year compared to ~1.5 years; whereas, females had greater VCO_2_ and RER levels and reduced VO_2_ levels at ~1.5 years compared to ~2 years (Table S2, Fig. 2). We observed significant sex effects on VO_2_ and VCO_2_ at all three time points, with females having greater values (*P* < 0.05, Table S2). There was only a significant sex effect on RER levels at ~2 years (*P* = 0.025, Table S2). Finally, there was a significant difference in RER levels at ~2 years compared to ~1 and ~1.5 years in both males and females (*P* < 0.01, Fig. 2).

**Table 2:** Effects of sex (male vs. female) and exercise (wheel vs. no wheel) on metabolic parameters. Measures were taken at ~1 year (prior to running wheel exposure), ~1.5, and ~2 years of age

Trait	*N*	Sex	Wheel access	Interaction	Activity	Batch
~Year 1						
VO_2_ (mL/kg/h)	59	*F*_1, 53_ = 189.696 ***P*** ** < 0.001**	*F*_1, 53_ = 0.036 *P* = 0.850	*F*_1, 53_ = 2.991 *P* = 0.090	*F*_1, 53_ = 2.614 *P* = 0.112	*F*_1, 53_ = 0.007 *P* = 0.936
VCO_2_ (mL/kg/h)	59	*F*_1, 53_ = 114.856 ***P*** ** < 0.001**	*F*_1, 53_ = 0.027 *P* = 0.651	*F*_1, 53_ = 0.264 *P* = 0.610	*F*_1, 53_ = 1.482 *P* = 0.229	*F*_1, 53_ = 0.144 *P* = 0.706
RER (VCO_2_/VO_2_)	59	*F*_1, 53_ = 2.642 *P* = 0.110	*F*_1, 53_ = 0.552 *P* = 0.461	*F*_1, 53_ = 1.486 *P* = 0.228	*F*_1, 53_ = 0.106 *P* = 0.746	*F*_1, 53_ = 0.049 *P* = 0.826
~Year 1.5						
VO_2_ (mL/kg/h)	44	*F*_1, 38_ = 93.906 ***P*** ** < 0.001**	*F*_1, 38_ = 1.390 *P* = 0.246	*F*_1, 38_ = 0.006 *P* = 0.939	*F*_1, 38_ = 1.553 *P* = 0.220	*F*_1, 38_ = 0.085 *P* = 0.772
VCO_2_ (mL/kg/h)	44	*F*_1, 38_ = 55.383 ***P*** ** < 0.001**	*F*_1, 38_ = 6.102 ***P*** ** = 0.018**	*F*_1, 38_ = 4.703 ***P*** ** = 0.036**	*F*_1, 38_ = 0.010 *P* = 0.921	*F*_1, 38_ = 0.070 *P* = 0.793
RER (VCO_2_/VO_2_)	44	*F*_1, 38_ = 2.863 *P* = 0.099	*F*_1, 38_ = 9.835 ***P*** ** = 0.003**	*F*_1, 38_ = 14.044 ***P*** ** = 0.001**	*F*_1, 38_ = 3.037 *P* = 0.089	*F*_1, 38_ = 0.041 *P* = 0.840
~Year 2						
VO_2_ (mL/kg/h)	45	*F*_1, 39_ = 5.351 ***P*** ** = 0.026**	*F*_1, 39_ = 0.446 *P* = 0.508	*F*_1, 39_ = 3.864 *P* = 0.056	*F*_1, 39_ = 7.405 ***P*** ** = 0.010**	*F*_1, 39_ = 1.054 *P* = 0.311
VCO_2_ (mL/kg/h)	45	*F*_1, 39_ = 9.370 ***P*** ** = 0.004**	*F*_1, 39_ = 0.038 *P* = 0.846	*F*_1, 39_ = 5.137 ***P*** ** = 0.029**	*F*_1, 39_ = 4.147 ***P*** ** = 0.049**	*F*_1, 39_ = 0.050 *P* = 0.824
RER (VCO_2_/VO_2_)	45	*F*_1, 39_ = 5.473 ***P*** ** = 0.025**	*F*_1, 39_ = 3.430 *P* = 0.072	*F*_1, 39_ = 1.521 *P* = 0.225	*F*_1, 39_ = 0.402 *P* = 0.530	*F*_1, 39_ = 3.071 *P* = 0.088

Statistical significance was judged at *P *<* *0.05 (in bold), and all *P*‐values presented are two‐tailed.

Food intake (as measured in the indirect calorimeters) was lower at ~1.5 years compared to ~1 year and greater at ~2 years compared to ~1.5 years in aging control mice. Water intake increased during aging in both sexes in the control cohorts. There were significant sex effects on food and water consumption at ~1 year and ~1.5 years (*P* < 0.005) and significant sex effects on water consumption at ~2 years (*P* = 0.001, Table 3). There were significant sex effects on home cage activity levels at all time points (*P* < 0.05). Female mice had greater levels of home cage activity compared to male mice (Table 3, Table S2).

**Table 3:** Effects of sex (male vs. female) and exercise (wheel vs. no wheel) on home cage activity. Measures were taken at ~1 year (prior to running wheel exposure), ~1.5, and ~2 years of age

Trait	Trans	*N*	Sex	Wheel access	Interaction	Batch
~Year 1						
Home cage activity		59	*F*_1, 54_ = 26.973	F_1, 54_ = 0.170	F_1, 54_ = 0.025	*F*_1, 54_ = 0.035
			***P*** ** < 0.001**	*P* = 0.682	*P* = 0.874	*P* = 0.852
Food consumption		59	*F*_1, 54_ = 15.459	*F*_1, 54_ = 0.055	*F*_1, 54_ = 0.416	*F*_1, 54_ = 0.281
			***P*** ** < 0.001**	*P* = 0.815	*P* = 0.521	*P* = 0.598
Water consumption	lg10	59	*F*_1, 54_ = 9.912	*F*_1, 54_ = 0.253	*F*_1, 54_ = 7.284	*F*_1, 54_ = 0.249
			***P*** ** = 0.003**	*P* = 0.617	***P*** ** = 0.009**	*P* = 0.620
~Year 1.5						
Home cage activity		44	*F*_1, 39_ = 6.070	*F*_1, 39_ = 1.166	*F*_1, 39_ = 10.402	*F*_1, 39_ = 9.860
			***P*** ** = 0.018**	*P* = 0.287	***P*** ** = 0.003**	***P*** ** = 0.003**
Food consumption		44	*F*_1, 39_ = 13.352	*F*_1, 39_ = 11.025	*F*_1, 39_ = 8.609	*F*_1, 39_ = 0.724
			***P*** ** = 0.001**	***P*** ** = 0.002**	***P*** ** = 0.006**	*P* = 0.400
Water consumption		43	*F*_1, 38_ = 10.837	*F*_1, 38_ = 9.618	*F*_1, 38_ = 0.792	*F*_1, 38_ = 4.231
			***P*** ** = 0.002**	***P*** ** = 0.004**	*P* = 0.379	***P*** ** = 0.047**
~Year 2						
Home cage activity		45	*F*_1, 40_ = 32.582	*F*_1, 40_ = 9.624	*F*_1, 40_ = 1.815	*F*_1, 40_ = 9.831
			***P*** ** < 0.001**	***P*** ** = 0.004**	*P* = 0.186	***P*** ** = 0.003**
Food consumption		45	*F*_1, 40_ = 3.232	*F*_1, 40_ = 5.452	*F*_1, 40_ = 1.869	*F*_1, 40_ = 1.858
			*P* = 0.080	***P*** ** = 0.025**	*P* = 0.179	*P* = 0.180
Water consumption		44	*F*_1, 39_ = 14.398	*F*_1, 39_ = 4.002	*F*_1, 39_ = 0.009	*F*_1, 39_ = 0.437
			***P*** ** = 0.001**	*P* = 0.052	*P* = 0.926	*P* = 0.512

Statistical significance was judged at *P *<* *0.05 (in bold), and all *P*‐values presented are two‐tailed.

### Long‐term physical activity varies during aging in a sex‐dependent manner

**Figure 3:**
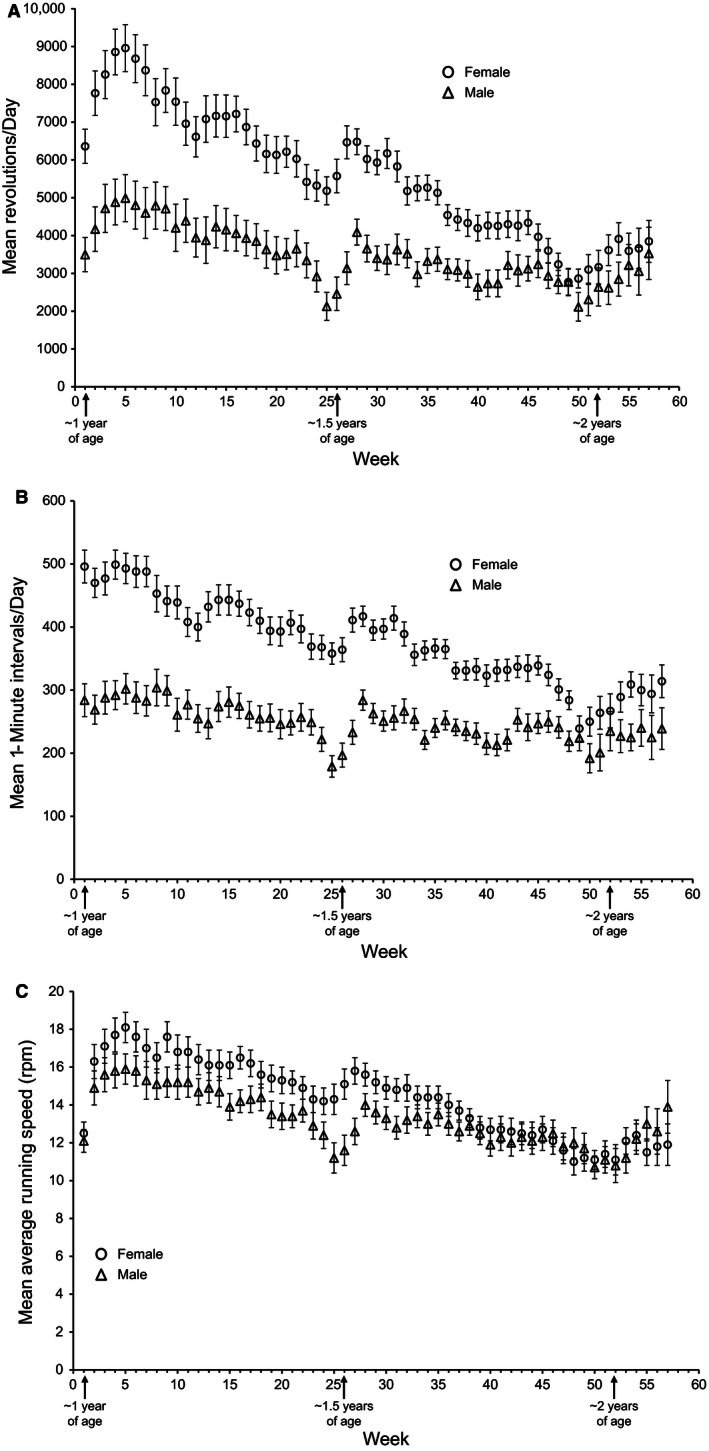
Estimated marginal means and standard errors of mean (A) revolutions per day, (B) time (i.e., cumulative 1 min intervals in which at least 1 revolution was recorded) spent running, and (C) running speed (mean revolutions/mean running time) across 57 weeks. (A) Comparisons between sexes by General Linear Models (GLM) revealed females ran significantly more than males during weeks 1–45. During weeks 46–57 there was no significant difference between the sexes. (C) Comparisons between sexes by GLM revealed females ran significantly faster only during weeks 15, 16, 25, 26, 27, and 31.

In the experimental cohorts of both sexes, we observed an increase in mean revolutions per day (distance) after mice gained wheel access (weeks 1 through 5 in Fig. 3A). Subsequently, distance run declined over the 57 weeks of wheel access. During weeks 25 and 51, mice were removed from their home cage and placed in metabolic cages (no wheel access) for 48 h and returned to their home cage after the metabolic measurements. After both metabolic analysis time points, there was an increase in distance run for several weeks, followed by a stabilization and decline (Fig. 3A). Exercise duration followed similar patterns as daily distance except there was no initial increase in duration over the first few weeks of wheel access (Fig. 3B). Mean average running speed (rpm) also followed similar patterns as distance (Fig. 3C). Thus, we observed an age‐related decline in all aspects of physical activity over the one‐year experimental period.

Females ran longer distances, for longer duration, and at higher speeds than males throughout the 57 weeks of wheel access. The sex effects were significant (*P* < 0.05) for distance from weeks 1–45 (~1 to ~1.85 years of age) of voluntary exercise. Similarly, significant sex effects on duration were observed during weeks 1–48 and week 54 (*P* < 0.05, Fig. 3B). There were very few weeks with significant sex effects on speed, although females had higher rpm than males and rpm slightly decreased with age in both sexes (Fig. 3C). Females demonstrated slightly different patterns of exercise in the early part of the study compared to males. Females had a sharper increase in distance (weeks 1–5) and a sharper decrease in distance (weeks 5–12) compared to male mice (Fig. 3A).

### Physical activity protects from age‐related metabolic changes

Overall body mass was significantly lower in experimental mice compared to control mice at all time points, with experimental mice having ~16% less body mass during ~ 1.1 to ~2 years (*P* < 0.05, Table 1, Fig. 1A, Table S1). Specifically, experimental mice followed similar temporal patterns of body mass changes as control mice but at significantly lower body mass. Female experimental mice had from 6 to 22 percent less body mass than female control mice. Male experimental mice had 11–19 percent less body mass than male control mice during aging (Table 1, Fig. 1A, Table S1).

Wheel access had a significant effect on body composition. Experimental mice had ~50% less body fat and ~15% more lean mass than control mice throughout the study. There were significant effects of exercise on percent fat and lean mass and on change in percent lean between experimental time points throughout the experiment (*P* < 0.001). Female experimental mice had 34–59% less percent body fat and 6–20% more percent lean mass than female control mice during aging from ~1.1 to ~2 years‐old. Thus, female mice with wheel access had ~52% less fat and ~16% more lean mass than control female mice from ~1.1 to 2 years. Male experimental mice had 44–55% less percent body fat and 9–17% more percent lean mass than male control mice during aging from ~1.1 to ~2 years. Male mice with wheel access had on average ~49% less body fat and ~15% more lean mass than control male mice from ~1.1 to ~2 years. There was only a significant wheel effect on change in percent fat observed between ~1 and ~1.1 years (*P* < 0.001). In the experimental cohort, female mice retained greater percentage lean mass than males until ~1.6 years old (Fig. 1C, B, Table 1, Table S1).

Wheel access only had a significant effect on VCO_2_ and RER at ~1.5 years (*P* < 0.04, Table 2, Table S2). RER levels in the experimental cohort increased with aging. RER levels in the experimental females increased from ~1 to ~1.5 years then remained stable from ~1.5 to ~2 years (Fig. 2).

Both food and water intake increased during aging in both sexes in the experimental cohort (Table S2). There was a significant wheel access effect on food and water consumption at ~1.5 years (*P* < 0.005) and on food consumption at ~2 years (*P* = 0.025, Table 3). Food and water intake was greater in experimental females than in experimental males. Both experimental males and females had greater food and water intake at both ~1.5 and ~2 years than control mice (Table S2). There was a slight decline in activity levels in control mice access during aging; whereas, experimental mice had an increase in home cage activity levels during aging but these effects were not significant except at ~2 years (*P* = 0.004, Table 3, Table S2).

### Exercise‐by‐sex interactions on metabolic changes during aging

We only observed a few significant exercise‐by‐sex interactions on metabolic changes during the aging process. The majority of the significant interactions on metabolic phenotypes collected by indirect calorimetry were observed at ~1.5 years. Significant interactions on percent change in mass and percent change in percent lean mass existed at ~1.4 and ~1.6 years (*P* < 0.05, Table 1, Fig. 1A). There was a significant exercise‐by‐sex interaction on RER levels observed at ~1.5 years and on VCO_2_ levels at ~1.5 and ~2 years. Females with wheel access had greater RER levels than control females at ~1.5 years; whereas males in both cohorts had the same RER levels at ~1.5 years (Fig. 1C, Table 2, Table S2).

### Phenotypic correlations between exercise and metabolic traits during aging

As expected, all physical activity phenotypes (distance, speed, time) were significantly and positively correlated at each time point during aging (Tables 4, 5). Body mass was significantly and positively correlated with percent fat and percent lean mass during aging. Distance and speed were significantly and negatively correlated with change in mass and change in percent lean mass at ~1.1 years (Table 5). VCO_2_ was significantly and positively correlated with VO_2_ and RER during aging. Exercise distance and time were significantly and positively correlated with RER levels at ~2 years but not at ~1 and ~1.5 years (Table 4). Body mass, percent fat, and percent lean mass were significantly correlated with RER at ~1 and ~1.5 years but not at ~2 years (Table 6). For a complete list of correlational analyses results see Tables 4, 5, 6.

**Table 4:** Pearson partial correlations for mean running traits during week 1, week 25, and week 51 of wheel access and metabolic traits measured immediately prior to each time point. Approximate age of individuals is one year, 1.5, and 2 years

Traits	Age		
	~1 year	~1.5 years	~2 years
Distance:Time	0.932^b^	0.943^b^	0.938^b^
Distance:Speed	0.793^b^	0.827^b^	0.706^b^
Distance:VO_2_	0.04	−0.097	−0.127
Distance:VCO_2_	−0.002	0.049	0.26
Distance:RER	−0.042	0.165	0.538^a^
Time:Speed	0.554^a^	0.661^a^	0.439^a^
Time:VO_2_	0.011	−0.067	−0.184
Time:VCO_2_	0.04	0.211	0.192
Time:RER	0.07	0.374	0.486^a^
Speed:VO_2_	0.075	−0.059	0.108
Speed:VCO_2_	−0.005	−0.058	0.314
Speed:RER	−0.095	−0.024	0.387
VO_2_:VCO_2_	0.837^b^	0.753^b^	0.745^a^
VO_2_:RER	0.088	0.162	0.037
VCO_2_:RER	0.613^b^	0.766^b^	0.691^b^

Pearson partial correlations (r; controlling for sex) are shown. Degrees of freedom = 26 for all pairwise comparisons at ~1 year, 20 at ~1.5 years, and 21 for ~2 years.

a *P* < 0.05.

b *P* < 0.001.

**Table 5:** Pearson partial correlations for mean running traits during week 1, 7, 21, 25, 33, 42, 51 of wheel access and body composition measured immediately prior to each time point

Traits	Age						
	~1 year	~1.1 years	~1.4 years	~1.5 years	~1.6 years	~1.8 years	~2 years
Distance:Time	0.923^b^	0.857^b^	0.942^b^	0.917^b^	0.918^b^	0.880^b^	0.930^b^
Distance:Speed	0.762^b^	0.877^b^	0.834^b^	0.804^b^	0.857^b^	0.834^b^	0.683^b^
Distance:Mass	−0.108	−0.366	−0.456^a^	−0.266	−0.24	−0.276	−0.166
Distance:% Fat	0.042	−0.266	−0.544^a^	−0.267	−0.338	−0.322	−0.232
Distance:% Lean	0.002	0.029	0.483^a^	0.272	0.293	0.271	0.109
Distance:% Δ in Mass	NA	−0.515^a^	−0.278	−0.122	0.023	−0.257	0.23
Distance:% Δ in %Fat	NA	−0.487^a^	−0.381	0.157	−0.088	−0.103	−0.004
Distance:% Δ in % Lean	NA	0.004	0.515^a^	0.412^a^	0.282	0.322	0.172
Time:Speed	0.492^a^	0.567^a^	0.642^b^	0.579^a^	0.605^a^	0.504^a^	0.39
Time:Mass	−0.15	−0.431^a^	−0.454^a^	−0.186	−0.205	−0.119	−0.026
Time:% Fat	0.005	−0.336	−0.529^a^	−0.128	−0.19	−0.049	−0.093
Time:% Lean	0.035	0.099	0.447^a^	0.118	0.143	0.006	−0.006
Time:% Δ in Mass	NA	−0.302	−0.157	−0.055	0.001	−0.097	0.308
Time:% Δ in %Fat	NA	−0.575^a^	−0.311	0.163	0.032	0.075	0.002
Time:% Δ in % Lean	NA	0.067	0.484^a^	0.235	0.161	0.067	0.04
Speed:Mass	−0.026	−0.356	−0.421^a^	−0.453^a^	−0.307	−0.412^a^	−0.396
Speed:% Fat	0.037	−0.352	−0.529^a^	−0.563^a^	−0.554^a^	−0.558^a^	−0.488^a^
Speed:% Lean	−0.001	0.126	0.479^a^	0.570^a^	0.525^a^	0.509^a^	0.383
Speed:% Δ in Mass	NA	−0.620^b^	−0.269	−0.05	0.105	−0.31	0.043
Speed:% Δ in %Fat	NA	−0.345	−0.311	0.167	−0.17	−0.225	−0.099
Speed:% Δ in % Lean	NA	0.126	0.511^a^	0.662^b^	0.494^a^	0.548^a^	0.445^a^
Mass:% Fat	0.752^b^	0.882^b^	0.824^b^	0.868^b^	0.800^b^	0.799^b^	0.739^b^
Mass:% Lean	−0.766^b^	−0.619^b^	−0.833^b^	−0.866^b^	−0.784^b^	−0.802^b^	−0.751^b^
Mass:% Δ in Mass	NA	0.16	0.463^a^	0.197	0.248	0.301	0.189
Mass:% Δ in %Fat	NA	0.332	0.12	0.056	0.084	0.238	0.148
Mass:% Δ in % Lean	NA	−0.706^b^	−0.774^b^	−0.743^b^	−0.783^b^	−0.817^b^	−0.777^b^
% Fat:% Lean	−0.986^b^	−0.700^b^	−0.792^b^	−0.950^b^	−0.981^b^	−0.992^b^	−0.969^b^
% Fat:% Δ in Mass	NA	0.224	0.403^a^	0.102	0.064	0.215	0.102
% Fat:% Δ in %Fat	NA	0.396^a^	0.419^a^	0.165	0.118	0.416^a^	0.497^a^
% Fat:% Δ in % Lean	NA	−0.791^b^	−0.797^b^	−0.821^b^	−0.946^b^	−0.990^b^	−0.989^b^
% Lean:% Δ in Mass	NA	−0.411^a^	−0.406^a^	−0.231	−0.136	−0.207	−0.091
% Lean:% Δ in %Fat	NA	−0.408^a^	−0.16	−0.117	−0.198	−0.398^a^	−0.527^a^
% Lean:% Δ in % Lean	NA	0.961^b^	0.890^b^	0.857^b^	0.950^b^	0.990^b^	0.989^b^
% Δ in Mass:% Δ in %Fat	NA	0.591^a^	0.567^a^	0.459^a^	0.653^b^	0.536^a^	0.202
% Δ in Mass:% Δ in % Lean	NA	−0.299	−0.33	−0.091	−0.123	−0.164	−0.069
% Δ in %Fat:% Δ in % Lean	NA	−0.257	−0.19	0.204	−0.099	−0.346	−0.444^a^

Pearson partial correlations (*r*; controlling for sex) are shown.

NA (Not Applicable) represents correlations that cannot be calculated since the measurements depend on a prior experimental time point.

a *P* < 0.05.

b *P* < 0.001.

**Table 6:** Pearson partial correlations for mean body composition traits and metabolic traits measured immediately prior to wheel access

Traits	Age		
	~1 year	~1.5 years	~2 years
Mass:% Fat	0.776^b^	0.911^b^	0.884^b^
Mass:% Lean	−0.763^b^	−0.894^b^	−0.879^b^
Mass:% Δ in Mass	NA	0.343^a^	0.567^b^
Mass:% Δ in % Fat	NA	0.108	0.401^a^
Mass:% Δ in % Lean	NA	−0.869^b^	−0.895^b^
Mass:VO_2_	−0.511^b^	−0.655^b^	−0.558^b^
Mass:VCO_2_	−0.632^b^	−0.600^b^	−0.512^a^
Mass:RER	−0.504^b^	−0.376^a^	−0.031
% Fat:% Lean	−0.984^b^	−0.991^b^	−0.986^b^
% Fat:% Δ in Mass	NA	0.320^a^	0.504^a^
% Fat:% Δ in % Fat	NA	0.181	0.580^b^
% Fat:% Δ in % Lean	NA	−0.921^b^	−0.973^b^
% Fat:VO_2_	−0.654^b^	−0.619^b^	−0.499^a^
% Fat:VCO_2_	−0.735^b^	−0.567^b^	−0.492^a^
% Fat:RER	−0.471^b^	−0.345^a^	−0.12
% Lean:% Δ in Mass	NA	−0.316^a^	−0.483^a^
% Lean:% Δ in % Fat	NA	−0.175	−0.602^b^
% Lean:% Δ in % Lean	NA	0.925^b^	0.974^b^
% Lean:VO_2_	0.644^b^	0.633^b^	0.478^a^
% Lean:VCO2	0.709^b^	0.549^b^	0.448^a^
% Lean:RER	0.436^b^	0.302^a^	0.053
% Δ in Mass:% Δ in % Fat	NA	0.528^b^	0.424^a^
% Δ in Mass:% Δ in % Lean	NA	−0.221	−0.480^a^
% Δ in Mass:VO2	NA	−0.299	−0.616^b^
% Δ in Mass:VCO2	NA	−0.25	−0.605^b^
% Δ in Mass:RER	NA	−0.109	−0.139
% Δ in % Fat:% Δ in % Lean	NA	0.064	−0.480^a^
% Δ in % Fat:VO2	NA	−0.036	−0.247
% Δ in % Fat:VCO2	NA	−0.088	−0.202
% Δ in % Fat:RER	NA	−0.121	0.041
% Δ in % Lean:VO2	NA	0.587^b^	0.508^a^
% Δ in % Lean:VCO2	NA	0.458^a^	0.487^a^
% Δ in % Lean:RER	NA	0.211	0.087
VO2:VCO2	0.850^b^	0.818^b^	0.923^b^
VO2:RER	0.229	0.320^a^	0.117
VCO2:RER	0.701^b^	0.799^b^	0.486^a^

Pearson partial correlations (*r*; controlling for sex, wheel access, and activity during metabolic measures) are shown.

NA (Not Applicable) represents correlations that cannot be calculated since the measurements depend on a pre‐experimental time point.

a *P* < 0.05.

b *P* < 0.001.

## Discussion

### Physical activity prevents age‐related, sex‐dependent changes in body mass and composition

The most interesting conclusion of this study is that regular long‐term exercise starting in midlife prevents body mass and body composition alterations observed during aging in both male and female mice. Mice that exercised had 16% lower body mass, 50% lower body fat, and 15% higher lean mass than mice who were not exposed to running wheels. The changes in body mass were not significantly correlated with specific physical activity measurements (distance, time, speed) indicating that engaging in exercise, and not the specific workout load, is potentially sufficient for maintenance of body weight and composition throughout aging. This may aid formulating recommendations of exercise in the aging human population, since it demonstrates that exercise even in reduced amount may be sufficient to alleviate age‐related changes in body mass and composition, at least in mice, although there could be a threshold of activity below which benefits are not achieved.

Changes in body mass and body composition during aging followed sex‐dependent trajectories in both experimental and control mice. It has been established that sex differences exist in physical activity levels, body mass, body composition, and metabolism in both humans and rodents (Lightfoot et al. 2004; Lightfoot 2008; Mauvais‐Jarvis 2015; Ackert‐Bicknell et al. 2016). In humans, cross‐sectional studies have suggested there are different trajectories of changes in body fat depending on sex (Hughes et al. 2002). Both longitudinal and cross‐sectional studies in humans have shown men and women gain weight specifically due to greater amount of fat than lean mass when less than 60 years old. Men older than 60 years display loss of weight and body fat. The trajectory for body composition changes in women after the age of 60 years has not been established (Hughes et al. 2002). Our findings demonstrate that there are sex differences in the trajectories of body mass and body composition changes during aging long‐term. In both sexes, introducing exercise midlife prevented accumulation of age‐related changes in body mass and composition. Even though work load declined during aging, participating in physical activity was still sufficient to protect against age‐related changes in body mass and composition in a sex‐dependent manner. There were significant exercise‐by‐sex interactions on changes in body mass and composition during aging, indicating exercise alters body mass and composition in a sex‐dependent manner. Interestingly, similar sex‐dependent trajectories of body mass and composition were observed in the experimental and control mice and could be attributed to the regulation of food intake in conjunction with increased activity. Food consumption is typically positively correlated with wheel running, but the extent of the effects can be sex dependent and contingent on initial body composition differences (Kelly et al. 2011). Future experiments, monitoring food consumption more regularly, are needed to determine the underlying causes of sex‐dependent trajectories of body mass and composition during aging.

### Physical activity levels change during aging in a sex‐dependent manner

Physical activity levels decline as humans age (Caspersen and Merritt 1995). The CDC surveillance data have found that 17% of adults 45–64 years‐old, 23% of adults 65–74 years‐old, and 36% of adults 75+ years‐old remain physically inactive (U.S. Department of Health and Human Services, 2008). In the current experiment, we observed a decline in exercise levels during voluntary participation over 12 months. There was an initial increase in distance and speed for the first 5 weeks of exercise followed by a decrease in physical activity. This also occurred after each indirect calorimetry measurement wherein mice were removed from the wheels for 48 h. These observations may extend from behavioral responses to a novel environment (new home cage, wheel access), adaption to the wheel, and potentially trained exercise ability. Novel environments induce stress response‐eliciting behavioral responses, such as increased activity to rewarding stimuli (de Visser et al. 2007; Novak et al. 2012; Nishijima et al. 2013). In particular, C57BL/6J mice respond to novel cage environment (with wheel access) by increased wheel activity (de Visser et al. 2007).

We observed a sex effect on physical activity, in which females ran greater distance and duration than males, consistent with previous reports (Leamy et al. 2009). These sex effects disappeared around ~46–49 weeks of exercise or ~23 months of age. However, after the initial 5 weeks of wheel access, females showed a sharp decline in physical activity and males showed a gradual decline in physical activity. There are several possible explanations for these declines. The decrease could be due to aging. Another possible explanation is loss of environmental novelty and habituation (Kolb et al. 2013). Lastly, decline in physical activity could result from reduced E2 levels. Acyclicity in rodents has been established as the menopausal transition, which occurs in human females during aging (Finch 2014). Although we do not have data for acyclicity, we would expect female mice to begin acyclicity at ~390–480 days (13–16 m) (Parkening et al. 1980; Nelson et al. 1982; Zarins et al. 2009). E2 contributes to regulating adipose development, exercise levels, and age‐related changes in body composition. Both human and animal studies have shown a decline in of E2 production causes insulin resistance, and exercise mitigates the resulting glucose intolerance and composition changes (Lightfoot 2008; Enns and Tiidus 2010; Kim 2014; MacDonald et al. 2015; Mauvais‐Jarvis 2015). Estrogen deficiency in postmenopausal women and ovariectomized rodents leads to higher RER levels, reduced lipid oxidation, and greater carbohydrate oxidation during rest and exercise (Zarins et al. 2009; Mauvais‐Jarvis 2015).

The decrease in physical activity levels, body mass changes, and percent fat changes in males that we observed during aging could also be due to hormonal changes. Reduction in testosterone levels during aging in males decreases fat‐free mass and increases body fat (Mudali and Dobs 2004). Additionally, physical activity levels are reduced in male rodents, including C57BL/6J, after castration (Lightfoot 2008). Hormonal changes during aging may also explain the sex‐dependent changes in physical activity levels, body mass, body composition, and metabolism that we observed.

### Changes in metabolism in response to aging and exercise

RER (ratio of CO2 production to O2 consumption) is used to indirectly determine relative use of carbohydrates or lipids for energy expenditure. Higher RER values (e.g. 1.0) indicate greater carbohydrate use; whereas lower RER values (e.g. 0.7) indicate greater lipid oxidation. Individuals, both rodents and humans, with sedentary lifestyles normally have higher RER values and lower fat oxidation. Physically active individuals tend to demonstrate lower RER values than untrained individuals in response to exercise (Ramos‐Jimenez et al. 2008; Mathes et al. 2011). Previous studies have shown that older C57BL/6J mice (660 days, ~1.8 years), compared to young mice (90 days), have greater body mass, reduced lean mass, VO_2_ and RER, greater FFAs, and lower triglycerides (Houtkooper et al. 2011). Sex differences in RER also exist. Females tend to store FFAs as triglycerides, which in turn assists in fat storage; whereas males oxidize circulating FFAs. Under increased energy demands from exercise, women oxidize a greater proportion of lipids versus carbohydrates than men, thus exercise results in lower RER in females (Zarins et al. 2009). We found that aging C57BL/6J mice had higher resting RER levels, especially at ~2 years, indicating a preference toward carbohydrate utilization in aged individuals. Most interestingly, female mice with wheel access had greater resting RER levels than control females. Thus, females utilize more carbohydrates as an energy source in response to exercise; whereas, male mice had no difference in RER levels in response to exercise.

We observed a decrease in both oxygen consumption (VO2) and carbon dioxide production (VCO2) during aging. Both VO2 consumption and VCO2 production are thought to be a proxy for the amount of metabolism occurring (e.g. more VO2 consumed and the more VCO2 produced the more metabolism occurring). We expected both VO2 and VCO2 to decrease during aging since metabolism and BMR are reduced with age (Roberts and Rosenberg 2006; Williams and Wood 2006; Herring et al. 2014). Although VO2 and VCO2 are used as proxies for metabolism, we cannot determine the efficiency of metabolism (e.g. the amount of ATP produced relative to oxygen intake) with only those measurements. We would expect VO2 to be positively correlated with running distance since we would expect a higher metabolism with greater running distance. It is possible the observed nonsignificant negative correlation of VO2 and running distance at 2 years of age could be due to a more efficient utilization of VO2 consumed in exercise‐trained mice.

In conclusion, this study demonstrates that exercise in mice protects against age‐related alterations in body mass, body composition and metabolism in both sexes. We showed that patterns of physiological changes during aging vary by sex. Additionally, sex impacts exercise abilities and physiological responses to exercise during aging. However, the benefits occur despite the significant differences in physiological and exercise ability between the sexes. We conclude that exposure to exercise from midlife on has significant benefits in mice that may extend to humans. Further studies need to determine if the benefits from long‐term exercise during aging occur due to exposure to exercise at a specific time and/or for specific duration of time. Future studies should investigate the effect of exercise in other outcomes such as cognitive function, cancer, heart disease, etc. Our results support the contention that laboratory mice are a valuable model to study the effects of age and exercise. In particular, the mouse could be used to add a genetic dimension to these studies, given the plethora of existing genetic resources in this organism.

## Conflicts of Interest

No conflicts of interest, financial or otherwise, are declared by the authors.

## Supporting information




**Table S1.** Estimated marginal means and standard errors corresponding to tests presented in Table 1.
**Table S2.** Estimated marginal means and standard errors corresponding to tests presented in Table 2 and 3.
