# Supplementary material for: Long‐term exercise in mice has sex‐dependent benefits on body composition and metabolism during aging
Source: Physiol Rep. 2016 Nov 14;4(21):e13011. doi: 10.14814/phy2.13011 (PMC5112492; doi:10.14814/phy2.13011)
Supplement: Supplementary file 1 — Table S1. Estimated marginal means and standard errors corresponding to tests presented in Table 1. Table S2. Estimated marginal means and standard errors corresponding to tests presented in Table 2 and 3. [file PHY2-4-e13011-s001.docx]

**Supporting Table 1.** Estimated marginal means and standard errors corresponding to tests presented in Table 1.

| **Trait** | **Female** | | | | **Male** | | | |
| --- | --- | --- | --- | --- | --- | --- | --- | --- |
|  | Control | | Experimental | | Control | | Experimental | |
|  | Mean | SE | Mean | SE | Mean | SE | Mean | SE |
| *~Year 1* |  |  |  |  |  |  |  |  |
| Body mass (g) | 26.1 | 0.6 | 25.0 | 0.6 | 34.5 | 0.6 | 33.8 | 0.6 |
| % Fat | 13.9 | 1.4 | 13.9 | 1.3 | 17.4 | 1.3 | 15.1 | 1.3 |
| % Lean | 79.7 | 1.3 | 79.8 | 1.2 | 76.5 | 1.2 | 79.4 | 1.2 |
| *~Year 1.1* |  |  |  |  |  |  |  |  |
| Body mass (g) | 26.1 | 0.7 | 24.2 | 0.7 | 37.3 | 0.7 | 33.0 | 0.7 |
| % Fat | 12.2 | 1.3 | 7.9 | 1.3 | 21.5 | 1.3 | 11.2 | 1.2 |
| % Lean | 81.0 | 1.3 | 85.8 | 1.2 | 73.2 | 1.2 | 82.0 | 1.2 |
| % Change in Mass | 1.5 | 1.4 | -3.3 | 1.3 | 6.9 | 1.3 | -1.4 | 1.2 |
| % Change in % Fat | -5.8 | 7.6 | -37.9 | 7.0 | 25.2 | 7.0 | -23.2 | 6.8 |
| % Change in % Lean | 83.1 | 2.0 | 89.9 | 1.9 | 72.3 | 1.9 | 84.2 | 1.8 |
| *~Year 1.4* |  |  |  |  |  |  |  |  |
| Body mass (g) | 31.9 | 1.1 | 25.8 | 0.9 | 41.2 | 0.9 | 35.4 | 0.9 |
| % Fat | 22.5 | 1.8 | 10.3 | 1.6 | 26.4 | 1.6 | 14.9 | 1.6 |
| % Lean | 70.9 | 1.8 | 82.5 | 1.6 | 67.6 | 1.6 | 78.7 | 1.5 |
| % Change in Mass | 19.5 | 1.5 | 5.5 | 1.4 | 10.1 | 1.3 | 7.3 | 1.3 |
| % Change in % Fat | 72.1 | 10.8 | 28.2 | 10.0 | 22.8 | 9.6 | 37.1 | 9.3 |
| % Change in % Lean | 71.1 | 3.1 | 87.1 | 2.8 | 62.6 | 2.7 | 82.2 | 2.6 |
| *~Year 1.5* |  |  |  |  |  |  |  |  |
| Body mass (g) | 33.3 | 1.2 | 26.2 | 1.0 | 42.3 | 1.0 | 35.8 | 1.0 |
| % Fat | 25.1 | 1.9 | 10.2 | 1.4 | 26.9 | 1.5 | 14.8 | 1.4 |
| % Lean | 68.4 | 1.7 | 81.5 | 1.3 | 66.9 | 1.4 | 78.4 | 1.3 |
| % Change in Mass | 1.9 | 0.9 | 1.2 | 0.7 | 2.8 | 0.7 | 1.1 | 0.7 |
| % Change in % Fat | 1.9 | 8.5 | 6.6 | 6.8 | 2.2 | 6.8 | 1.0 | 6.6 |
| % Change in % Lean | 62.6 | 3.4 | 86.6 | 2.7 | 59.1 | 2.7 | 80.1 | 2.6 |
| *~Year 1.6* |  |  |  |  |  |  |  |  |
| Body mass (g) | 33.0 | 1.3 | 26.5 | 1.0 | 42.1 | 1.1 | 34.2 | 1.0 |
| % Fat | 23.2 | 2.2 | 11.0 | 1.7 | 25.3 | 1.7 | 11.4 | 1.7 |
| % Lean | 71.9 | 2.0 | 82.4 | 1.6 | 70.5 | 1.6 | 82.7 | 1.6 |
| % Change in Mass | -0.7 | 1.4 | 1.4 | 1.0 | -0.8 | 1.1 | -4.6 | 1.0 |
| % Change in % Fat | -9.8 | 5.8 | 10.0 | 4.5 | -7.0 | 4.6 | -20.7 | 4.5 |
| % Change in % Lean | 67.3 | 3.8 | 88.3 | 2.9 | 64.1 | 3.0 | 86.2 | 2.9 |
| *~Year 1.8* |  |  |  |  |  |  |  |  |
| Body mass (g) | 33.6 | 1.6 | 27.4 | 1.1 | 40.7 | 1.1 | 34.1 | 1.1 |
| % Fat | 27.6 | 2.5 | 12.5 | 1.7 | 22.7 | 1.8 | 10.2 | 1.7 |
| % Lean | 69.2 | 2.3 | 81.2 | 1.6 | 72.8 | 1.6 | 84.2 | 1.6 |
| % Change in Mass | -2.2 | 1.9 | 3.2 | 1.3 | -3.2 | 1.4 | -0.3 | 1.3 |
| % Change in % Fat | 1.0 | 6.9 | 13.5 | 4.7 | -12.7 | 4.9 | -12.1 | 4.7 |
| % Change in % Lean | 60.9 | 4.1 | 85.2 | 2.8 | 65.8 | 2.9 | 87.5 | 2.8 |
| *~Year 2* |  |  |  |  |  |  |  |  |
| Body mass (g) | 35.1 | 1.9 | 27.4 | 1.3 | 37.7 | 1.3 | 33.5 | 1.4 |
| % Fat | 28.6 | 2.8 | 12.6 | 1.9 | 17.3 | 2.0 | 8.7 | 2.0 |
| % Lean | 67.0 | 2.6 | 80.7 | 1.8 | 77.8 | 1.8 | 85.1 | 1.9 |
| % Change in Mass | 4.3 | 2.0 | -0.1 | 1.3 | -7.8 | 1.4 | -1.2 | 1.4 |
| % Change in % Fat | 3.6 | 8.7 | 3.6 | 5.9 | -28.6 | 6.2 | -22.1 | 6.3 |
| % Change in % Lean | 55.9 | 4.5 | 83.9 | 3.0 | 74.0 | 3.2 | 88.2 | 3.2 |

| **Trait** | **Trans** | **Female** | | | | **Male** | | | |
| --- | --- | --- | --- | --- | --- | --- | --- | --- | --- |
|  |  | Control | | Experimental | | Control | | Experimental | |
|  |  | Mean | SE | Mean | SE | Mean | SE | Mean | SE |
| *~Year 1* |  |  |  |  |  |  |  |  |  |
| VO_2_ (ml/kg/h) |  | 3423.5 | 74.5 | 3326.5 | 47.9 | 2537.9 | 56.3 | 2600.9 | 68.6 |
| VCO_2_ (ml/kg/h) |  | 2820.5 | 86 | 2840 | 55.2 | 2069.7 | 64.9 | 2144 | 79.1 |
| RER (VCO_2_/VO_2_) |  | 0.82 | 0.02 | 0.85 | 0.01 | 0.82 | 0.01 | 0.82 | 0.01 |
| Home Cage Activity |  | 2501.9 | 185 | 2391.4 | 113.9 | 1802.7 | 139.7 | 1728.5 | 165.2 |
| Food Consumption |  | 3.83 | 0.34 | 3.79 | 0.21 | 2.73 | 0.26 | 2.97 | 0.31 |
| Water Consumption | lg10 | 0.578 | 0.043 | 0.532 | 0.026 | 0.41 | 0.032 | 0.508 | 0.038 |
| *~Year 1.5* |  |  |  |  |  |  |  |  |  |
| VO_2_ (ml/kg/h) |  | 3042.4 | 110 | 3174.5 | 60.1 | 2152.3 | 82.1 | 2295 | 83.6 |
| VCO_2_ (ml/kg/h) |  | 2409.8 | 136.7 | 2954.5 | 74.7 | 1751.9 | 102 | 1922.9 | 103.9 |
| RER (VCO_2_/VO_2_) |  | 0.78 | 0.03 | 0.93 | 0.01 | 0.81 | 0.02 | 0.83 | 0.02 |
| Home Cage Activity |  | 2021.4 | 194.1 | 1931.7 | 132.1 | 1332.5 | 146.1 | 1983.1 | 184.8 |
| Food Consumption |  | 2.88 | 0.49 | 5.88 | 0.33 | 2.54 | 0.37 | 3.85 | 0.46 |
| Water Consumption |  | 2.95 | 0.4 | 4.8 | 0.27 | 2.28 | 0.31 | 3.71 | 0.37 |
| *~Year 2* |  |  |  |  |  |  |  |  |  |
| VO_2_ (ml/kg/h) |  | 2909.4 | 164.8 | 2959.9 | 117.7 | 2704.8 | 131.7 | 2410.9 | 131.8 |
| VCO_2_ (ml/kg/h) |  | 2562.4 | 162.7 | 2793.4 | 116.2 | 2266.1 | 130 | 2105.2 | 130.1 |
| RER (VCO_2_/VO_2_) |  | 0.88 | 0.02 | 0.93 | 0.02 | 0.84 | 0.02 | 0.87 | 0.02 |
| Home Cage Activity |  | 2097 | 188.6 | 2633.6 | 99.6 | 1279.1 | 132.1 | 2103 | 150.4 |
| Food Consumption |  | 3.67 | 0.46 | 5.27 | 0.24 | 3.51 | 0.32 | 4.4 | 0.37 |
| Water Consumption |  | 3.96 | 0.4 | 4.86 | 0.21 | 2.99 | 0.29 | 3.93 | 0.31 |

**Supporting Table 2**. Estimated marginal means and standard errors corresponding to tests presented in Table 2 and 3.
